# Prevalence and determinants of socioeconomic inequality in caesarean section deliveries in Bangladesh: an analysis of cross-sectional data from Bangladesh Demographic Health Survey, 2017-18

**DOI:** 10.1186/s12884-023-05782-4

**Published:** 2023-07-04

**Authors:** Pradeep Kumar, Himani Sharma

**Affiliations:** 1 Monitoring & Evaluation, Health Action Trust, Lucknow, India; 2 Department of Survey Research and Data Analytics, International Institute for Population Sciences, Deonar, Mumbai, 400088 India

**Keywords:** Caesarean, Socio-economic inequality, Decomposition, Bangladesh

## Abstract

**Background:**

Caesarean section deliveries, which involve incisions in the abdomen and uterus of the mother, have been a widespread event among women with obstructed labour. The current study not only estimated the socioeconomic and demographic factors of caesarean deliveries in Bangladesh but also decomposed the existing socioeconomic inequality in caesarean deliveries.

**Data and methods:**

2017-18 Bangladesh Demographic and Health Survey (BDHS) data was used for this study. The adequate sample size for the analysis was 5,338 women aged 15–49 years who had given birth at a health facility for three years preceding the survey. Explanatory variables included women’s age, women’s educational level, women’s working status, mass media exposure, body mass index (BMI), birth order, Ante Natal Care (ANC) visits, place of delivery, partner’s education and occupation, religion, wealth index, place of residence, and divisions. Descriptive statistics along with bivariate and multivariate logistic regression analysis was performed to identify the factors associated with the outcome variable. Concentration index and concentration curve were made to measure the socioeconomic inequality in caesarean births in Bangladesh. Further, Wagstaff decomposition analysis was used to decompose the inequalities in the study.

**Results:**

About one-third of the deliveries in Bangladesh were caesarean. Education of the women and the family’s wealth had a positive relationship with caesarean delivery. The likelihood of caesarean delivery was 33% less among working women than those who were not working [AOR: 0.77; CI: 0.62–0.97]. Women who had mass media exposure [AOR: 1.27; CI: 0.97–1.65], overweight/obese [AOR: 1.43; CI: 1.11–1.84], first birth order, received four or more Antenatal check-ups (ANC) [AOR: 2.39; CI: 1.12–5.1], and delivered in a private health facility [AOR: 6.69; CI: 5.38–8.31] had significantly higher likelihood of caesarean delivery compared to their counterparts. About 65% of inequality was explained by place of delivery followed by wealth status of the household (about 13%). ANC visits explained about 5% of the inequality. Furthermore, the BMI status of the women had a significant contribution to caesarean births-related inequality (4%).

**Conclusion:**

Socioeconomic inequality prevails in the caesarean deliveries in Bangladesh. The place of delivery, household wealth status, ANC visits, body mass index, women’s education and mass media have been the highest contributors to the inequality. The study, through its findings, suggests that the health authorities should intervene, formulate specialized programs and spread awareness about the ill effects of caesarean deliveries amongst the most vulnerable groups of women in Bangladesh.

## Background

Caesarean section (C-section) deliveries, which involve incisions in the abdomen and uterus of the mother, have been a widespread event among women with obstructed labour [1]. C-section deliveries prove to be a lifesaving intervention for both the mother and her unborn baby. However, in recent times, the prevalence of caesarean deliveries has been manifold across the globe [2]. According to the World Health Organization (WHO), the ideal rate for caesarean deliveries should be between 5 and 15%; thereby, a rate higher than 15% signifies overuse [3]. A significant variation is observed in the rates of caesarean deliveries in high and low-income countries [4]. The rates of caesarean section delivery have risen from 7% to 1990 to 21% today across the world and are projected to increase even more over time. According to a study by World Health Organization, if this trend continues, by 2030, the highest rates are likely to be in Eastern Asia (63%), Latin America and the Caribbean (54%), Western Asia (50%), Northern Africa (48%) Southern Europe (47%) and Australia and New Zealand (45%). This number is set to increase over the coming decade, with nearly a third (29%) of all births are likely to take place through caesarean section by 2030, the research finds [5].

A study conducted in 2016 showed that one in five women in the world deliver by CS. The study further revealed that presently, 40% of all births are by CS in Latin America while Caribbean and Southern America are the sub regions with the highest rates of CS in the world with 42%. Africa shows the lowest average rate of CS with 7%, which is a weighted average between 3.5% in sub-Saharan Africa and 27% in Northern Africa [6]. A study based on the Demographic Health Survey data of nine developing countries of South and South-east Asia, viz., Vietnam, India, Maldives, Timor-Leste, Nepal, Indonesia, Pakistan, Bangladesh and Cambodia, revealed a significant inclination toward the institutional deliveries in these countries [7]. In Bangladesh itself, deliveries from C-sections have tremendously increased from 4 to 23% in last 10 years [3]. A meta-analysis study including Nepal, Bangladesh, Pakistan, and India showed that caesarean section deliveries are increasing in all four countries [8]. Cesarean section delivery is more prevalent among mothers of older age groups and women residing in urban and affluent areas [9]. Previously conducted studies on C-section have indicated that CS were associated positively with advanced maternal age and obesity [10]. A multi country analysis study based on data from over 20,000 births revealed that the women of higher socio-economic background, who had better access to antenatal services are the most likely to undergo a caesarean section in Bangladesh, Colombia, Dominican Republic, Egypt, Morocco and Vietnam. [11].

C-section deliveries are unforeseeable and certainly have helped many women deliver the babies ensuring their safety and wellbeing. However, the identification of the cases in which there is a need to incorporate the C-section deliveries needs to be considered [12]. With this rate of increasing C-section deliveries, it is crucial to identify the C-section factors to lessen the interventions and provide it to the cases that require it the most [8, 13]. To ensure this, the Robson classification can be used as a standard way of screening genuine cases of caesarean deliveries based on five parameters such as obstetric history, the onset of labour, fetal presentation or lie, number of neonates and gestational age [14].

Caesarean deliveries lead to many problems, such as post-delivery risks, the financial burden on the family, and profound brain damage among babies. They can be catastrophic for caregivers and family members [15]. Differences in caesarean rates between public and private healthcare facilities are also important to understand. Studies have shown that even in the underserved areas in South Asia, particularly in Bangladesh, caesarean deliveries are greater in private facilities [16].

Bangladesh has made tremendous efforts in reducing the gaps in maternal health care services over the past few years. Nonetheless, the instances of prevalent socioeconomic inequality in several indicators of maternal healthcare services in Bangladesh are well documented [17, 18]. The findings from studies concerning South Asian countries like Nepal and Pakistan disclose that facility delivery is mostly determined by the social determinants of health rather than the individual health risk and horizontal inequities in the use of facility-based delivery services do favor the better off than the poorer individuals [19]. A comprehensive study of universal health coverage in Bangladesh with 19 nationally representative population based surveys revealed that the existing socioeconomic inequality in delivery care services is projected to continue until 2030. It suggested that most of the included health services were more concentrated among the wealthier poorer households in comparison to the poorer ones [20]. Not just Bangladesh, studies based on diverse geographical and cultural settings of south Asia and Sub-Saharan Africa also portray a similar picture. Health inequalities across countries like Ethiopia, Nepal and Zimbabwe have often been observed with reference to utilization of antenatal care, institutional delivery and postnatal care [21]. In Nigeria too, inequality in the use of health care services was found, favoring the advantaged women irrespective of the existing maternal health promotion initiatives in the country [22]. So far, many studies have reported the risk factors of C-section delivery in Bangladesh [2, 23–25]. Nevertheless, most of these studies estimated the socioeconomic and demographic predictors that may affect the C-section deliveries. A few earlier studies on Bangladesh have stated that inequality in delivery care and C-section deliveries was highly associated with wealth status. These studies were conducted quite a time ago, and there is a need to identify the level of inequality in the current scenario [18]. Moreover, there has been no significant improvement in reducing socioeconomic inequality in the delivery of care services in the recent time in Bangladesh. Given that there are inequities in the prevalence of CS within countries, it is typical to focus on monitoring these to improve maternal health [26]. The current study, contributing in this regard, not only estimated the socioeconomic and demographic factors of caesarean deliveries in Bangladesh but also decomposed the existing socioeconomic inequality in C-section deliveries. Moreover, this study uses the most recent Bangladesh demographic and health survey to show the determinants of C-section deliveries and socioeconomic inequality.

## Data and methods

### Data source

The study was carried out on the 2017-18 Bangladesh Demographic and Health Survey (BDHS) data, a nationally representative survey of 20,127 women aged 15–49 years from 19,457 households covering 672 sample points (clusters) from both urban and the rural areas throughout Bangladesh. The BDHS was conducted by the National Institute for Population Research and Training (NIPORT) of the Ministry of Health and Family Welfare [27]. The 2017-18 survey used a two-stage stratified sample of the households. Bangladesh Census was used as a sampling frame from the enumeration areas (EAs) of the 2011 Population and Housing Census of the People’s Republic of Bangladesh, provided by the Bangladesh Bureau of Statistics (BBS). Bangladesh has a five tier government system, starting from Divisions at Level 1, followed by districts at Level 2, sub districts at Level 3, Union council, municipalities and city corporations at level 4 and villages and wards at Level 5. The primary sampling unit (PSU) of the survey is an EA with an average of about 120 households. In the first stage, 675 EAs (250 in urban areas and 425 in rural areas) were selected with probability proportional to EA size. In the second stage of sampling, a systematic sample of an average of 30 households per EA was selected to provide statistically reliable estimates of key demographic and health variables for the country, for urban and rural areas separately [27]. The BDHS obtained detailed information on fertility levels, marriage, fertility preferences, awareness and use of family planning methods, breastfeeding practices, nutritional status of women and young children, childhood mortality, maternal and child health, and knowledge and attitudes regarding HIV/AIDS and other sexually transmitted infections. The detailed information on the survey is given elsewhere [27]. The adequate sample size for the analysis was 5,338 women aged 15–49 years who had given birth at a health facility for three years preceding the survey.

### Variable description

#### Outcome variable

The outcome variable for the study was caesarean section deliveries among women aged 15–49 years. The women were asked, “was the baby delivered by caesarean section? Did they cut your belly open to take the baby out?“ The response was coded as 0 “no” and 1 “yes”.

#### Explanatory variables

These included women’s age (15–24, 25–34, and ≥ 35 years), women’s educational level (no education, primary, secondary, and higher), working status (not working and working), body mass index (BMI) (underweight: <18.5 kg/m^2^, normal: 18.5–24.9 kg/m^2^, and overweight/obese: ≥25 kg/m^2^), birth order (first, second, three or more), Ante Natal Care (ANC) visits (no visit, 1–3 visit, and four or more), place of delivery (public and private), partner’s education (no education, primary, secondary, and higher), partner’s occupation (unemployed, professional/technical/managerial, sales, agricultural, services, and skilled and unskilled), religion (Islam and others), wealth index (poorest, poorer, middle, richer, and richest), place of residence (urban and rural), divisions (Barisal, Chittagong, Dhaka, Khulna, Mymensingh, Rajshahi, Rangpur, and Sylhet) and mass media exposure (Yes and No). Exposure to mass media was captured by asking the respondents about the frequency of reading newspaper/ magazine; listening to radio and watching television. These explanatory variables have been used for univariate, bivariate, logistic regression model and inequality analysis in the study.

### Statistical analysis

Descriptive statistics were used to observe the distribution of the study population. Further, bivariate and multivariate logistic regression analysis was performed to identify the factors associated with the outcome variable. A chi-square test was performed to examine the association between women’s background characteristics and caesarean birth along with a binary logistic regression computing the adjusted odds ratio (AOR) as well. Additionally, sensitivity analysis along with multilevel modelling was done using robust standard errors.

### Concentration index

In BDHS, the wealth quintile was the key variable to measure the household’s economic status. The wealth index was generated through household scores based on the ownership of consumer and household goods and amenities such as toilet facilities, sources of drinking water, and flooring material. These scores were used to classify all households into wealth quantiles. The lowest quantile has the poorest 20%, while the uppermost quantile has the wealthiest 20%. Afterwards, this wealth score was used for decomposition analysis and Concentration index (CCI) calculation. This helped us to calculate the concentration index and concentration curve (CC) to measure the socioeconomic inequality in caesarean births in Bangladesh. The concentration index represents the magnitude of inequality by measuring the area between the concentration curve and line of equality and calculated as twice the weighted covariance between the outcome and fractional rank in the wealth distribution divided by the variable mean.

The concentration index can be written as follows:

$$\varvec{C}=\frac{2}{\varvec{\mu }}\varvec{c}\varvec{o}\varvec{v}\left({\varvec{y}}_{\varvec{i},}{\varvec{R}}_{\varvec{i}}\right)$$

Where C is the concentration index; *y*_*i*_ is the outcome variable index; ***R*** is the fractional rank of individual ***i*** in the distribution of socioeconomic position; $\varvec{\mu }$ is the mean of the outcome variable of the sample, and $\varvec{c}\varvec{o}\varvec{v}$ denotes the covariance [28]. The index value lies between − 1 to + 1.

If the curve lies above the line of equality, the concentration index takes a negative value, indicating a disproportionate concentration of inequality among the poor (pro-rich). Conversely, if the curve lies below the line of equality, the concentration index takes a positive value, indicating a disproportional concentration of inequality among the rich (pro-poor). In the absence of socioeconomic related inequality, the concentration index is zero. The value of CCI quantifies the extent of socioeconomic inequality. The larger the absolute value, the greater the inequalities.

#### Decomposition of the concentration index

The study used Wagstaff decomposition analysis to decompose the concentration index. Wagstaff’s decomposition demonstrated that the concentration index could be decomposed into the contributions of each factor to the income-related inequalities [29]. Each contribution is the outcome of the sensitivity of health concerning that socioeconomic factor and the extent of income-related inequality in that factor. Based on the linear regression relationship between the outcome variable ${y}_{i}$, the intercept α, the relative contribution of ${x}_{ki}$ and the residual error ${\epsilon }_{i}$

$${y}_{i}=\alpha +\sum {\beta }_{k}{x}_{ki}+{\epsilon }_{i}$$

Where ${\epsilon }_{i}$ is an error term, given the relationship between y and ${x}_{i}$, the CCI for y (C) can be rewritten as

$$C=\sum \left(\frac{{\beta }_{k}{\stackrel{-}{x}}_{k}}{\mu }\right){C}_{k}+\frac{GC\epsilon }{\mu }/\mu$$

Where $\mu$ is the mean of ${ y}_{i}$, ${\stackrel{-}{x}}_{k}$ is the mean of ${x}_{k}$, ${\beta }_{k}$ is the coefficient from a linear regression of outcome variable, ${C}_{k }$ is the concentration index for ${x}_{k}$ (defined analogously to C, and GC_ε_ is the generalized concentration index for the error term (${\epsilon }_{i}$).

Here, C is the outcome of two components: First, the determinants or ‘explained’ factors, which are equivalent to the weighted accumulation of the concentration indices of the regressor, where one unit change in the outcome variable is to be associated with the one unit change in the explanatory variable. The explained factors indicate that the proportion of inequalities in the outcome variable is explained by the selected explanatory factors, i.e., x_k_. Second, a residual or ‘unexplained’ factor$\left(\frac{GC\epsilon }{\mu }/\mu \right)$, indicates the inequality in health variables that cannot be explained by selected explanatory factors across various socioeconomic groups [30].

## Results

### Sample distribution of study population, Bangladesh, BDHS, 2017-18

**Fig. 1:**
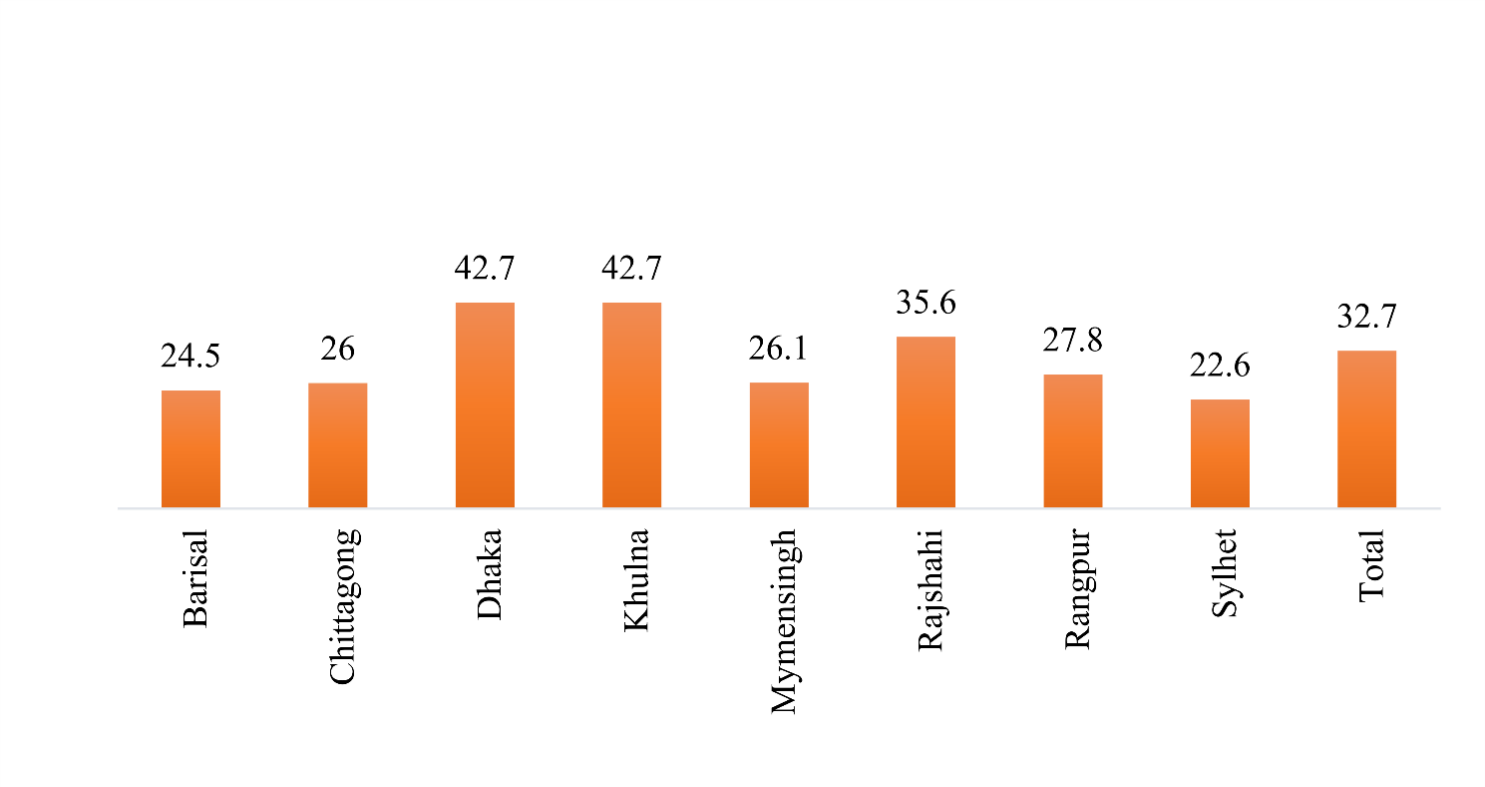
Prevalence of caesarean section deliveries among women in Bangladesh and its divisions, Bangladesh Demographic Health Survey, 2017-18.

Table 1 presents the sociodemographic profile of respondents in the study. More women belonged to the 15–24 years of age group (53.1%), 17% had higher education, and about 37% were working. More than half of the women had mass media exposure, 18% were underweight, and 22% were overweight/obese. About 40% of women had first birth order, 47% received four or more ANC, and 35% of women delivered at a private facility. Around 18% of partners had higher education, and only 19% of women’s husbands were in services. Figure 1 presents the prevalence of caesarean section deliveries among women in Bangladesh. The national prevalence of caesarean section deliveries in Bangladesh was 32% wherein the divisions of Dhaka and Khulna showed the highest prevalence (42.7%) while Sylhet showed the lowest prevalence of caesarean deliveries (22.6%).

**Table 1:** Sample distribution of women aged 15-49 in Bangladesh by various background characteristics, Bangladesh Demographic Health Survey, 2017-18

Variables	Sample	Percentage (95% confidence interval)
**Women’s age (in years)**		
15–24	2,836	53.1 (51.8–54.5)
25–34	2,198	41.2 (39.9–42.5)
≥35	304	5.7 (5.1–6.4)
**Women’s education**		
No education	351	6.6 (5.9–7.3)
Primary	1,471	27.6 (26.4–28.8)
Secondary	2,609	48.9 (47.5–50.2)
Higher	906	17.0 (16.0–18.0)
**Working status**		
No	3,365	63.0 (61.7–64.3)
Yes	1,973	37.0 (35.7–38.3)
**Mass Media Exposure**		
No Exposure	1,854	34.7 (33.5–36.0)
Exposure	3,484	65.3 (64.0–66.5)
**Body Mass Index**		
Underweight	804	17.6 (16.6–18.8)
Normal	2,746	60.2 (58.8–61.7)
Overweight and Obese	1,009	22.1 (20.9–23.4)
**Birth order**		
First	2,081	39.0 (37.7–40.3)
2nd order	1,715	32.1 (30.9–33.4)
3 or more	1,542	28.9 (27.7–30.1)
**ANC visits**		
No visit	405	8.0 (7.3–8.8)
1–3 visits	2,272	45.0 (43.6–46.4)
4 or more	2,374	47.0 (45.6–48.4)
**Place of delivery**		
Public	763	14.3 (13.4–15.3)
Private	1,887	35.3 (13.4–15.3)
**Husband’s education**		
No education	802	15.0 (14.1–16.0)
Primary	1,789	33.5 (32.3–34.8)
Secondary	1,778	33.3 (32.1–34.6)
Higher	969	18.2 (17.1–19.2)
**Husband’s occupation**		
Unemployed	118	2.2 (1.8–2.6)
Professional/Technical/Managerial	432	8.1 (7.4–8.9)
Sales	969	18.2 (17.1–19.2)
Agricultural	1,008	18.9 (17.9–20.0)
Services	712	13.4 (12.5–14.3)
Skilled And Unskilled	2,099	39.3 (38.0–40.6)
**Religion**		
Islam	4,910	92.0 (91.2–92.7)
Others	428	8.0 (7.3–8.8)
**Wealth Index**		
Poorest	1,108	20.8 (19.7–21.9)
Poorer	1,106	20.7 (19.7–21.8)
Middle	1,020	19.1 (18.1–20.2)
Richer	1,071	20.1 (19.0–21.2)
Richest	1,034	19.4 (18.3–20.4)
**Place of residence**		
Urban	1,427	26.7 (25.6–27.9)
Rural	3,911	73.3 (72.1–74.4)
**Division**		
Barisal	303	5.7 (5.1–6.3)
Chittagong	1,141	21.4 (20.3–22.5)
Dhaka	1,359	25.5 (24.3–26.6)
Khulna	481	9.0 (8.3–9.8)
Mymensingh	451	8.4 (7.7–9.2)
Rajshahi	622	11.7 (10.8–12.5)
Rangpur	555	10.4 (9.6–11.3)
Sylhet	425	8.0 (7.3–8.7)
**Total**	**5,338**	**100.0**

Table 2 shows that the likelihood of caesarean delivery was twice among women aged 35 years and above than those who belonged to 15–24 years’ age group [AOR: 2.06; CI: 1.19–3.55]. Education of the women had a positive relationship with caesarean delivery. For instance, the prevalence of caesarean birth increases with the increase of the women’s level of education. The likelihood of caesarean delivery was 33% less likely among working women than those who were not working [AOR: 0.77; CI: 0.62–0.97]. Moreover, women who had mass media exposure were 27% more likely to have caesarean births than those who did not [AOR: 1.27; CI: 0.97–1.65]. The likelihood of caesarean delivery was 43% more likely among women who were overweight/obese than those who had normal BMI [AOR: 1.43; CI: 1.11–1.84]. First birth order women reported more caesarean delivery than women who had three or more births. Women who received four or more ANC had 2.39 times more odds of caesarean delivery than those who had no ANC visit [AOR: 2.39; CI: 1.12–5.1]. Moreover, women who delivered in a private health facility were 6.69 times more likely to have a caesarean birth than those delivered in a public health facility [AOR: 6.69; CI: 5.38–8.31]. Similar to education, the family’s wealth also had a positive association with caesarean birth among women. With reference to women who belonged to the poorest families, women who belonged to the wealthiest families had an 84% higher chance of caesarean birth [AOR: 1.84; CI: 1.15–2.95]. The prevalence of caesarean births was significantly higher in the divisions, Dhaka and Khulna.

**Table 2:** Prevalence of caesarean delivery and odds ratio for risk of caesarean birth by background characteristics, Bangladesh Demographic Health Survey, 2017-18

Background characteristics	C-Section	p-value	AOR [95% CI]
**Women’s age (in years)**		0.586	
15–24	33.1		
25–34	32.5		1.17(0.89–1.54)
≥35	31.2		2.06**(1.19–3.55)
**Women’s education**		< 0.0001	
No education	16.4		
Primary	17.9		0.58*(0.31–1.09)
Secondary	34.0		0.64(0.35–1.17)
Higher	59.4		0.72(0.37–1.41)
**Working status**		< 0.0001	
No	37.3		
Yes	25.0		0.77**(0.62–0.97)
**Mass Media Exposure**		< 0.0001	
No Exposure	17.0		
Exposure	41.1		1.27*(0.97–1.65)
**Body Mass Index**		< 0.0001	
Underweight	25.7		0.98(0.73–1.31)
Normal	29.1		
Overweight and Obese	50.2		1.43***(1.11–1.84)
**Birth order**		< 0.0001	
First	40.2		
2nd order	34.4		1.06(0.81–1.39)
3 or more	20.8		0.72*(0.51–1.04)
**ANC visits**		< 0.0001	
No visit	6.0		
1–3 visits	23.7		1.66(0.78–3.52)
4 or more	46.8		2.39**(1.12–5.1)
**Place of delivery**		< 0.0001	
Public	35.1		
Private	78.4		6.69***(5.38–8.31)
**Husband’s education**		< 0.0001	
No education	18.2		
Primary	22.1		0.73(0.49–1.1)
Secondary	35.4		0.76(0.5–1.16)
Higher	59.7		0.93(0.57–1.5)
**Husband’s occupation**		< 0.0001	
Unemployed	32.0		
Professional/Technical/Managerial	62.1		1.30(0.61–2.79)
Sales	37.6		1.16(0.57–2.35)
Agricultural	20.8		1.33(0.64–2.75)
Services	34.8		1.53(0.74–3.15)
Skilled And Unskilled	29.5		1.13(0.57–2.26)
**Religion**		0.022	
Islam	32.2		
Others	38.5		0.94(0.67–1.31)
**Wealth Index**		< 0.0001	
Poorest	13.0		
Poorer	22.3		1.25(0.85–1.85)
Middle	30.8		1.22(0.81–1.83)
Richer	38.1		1.09(0.72–1.64)
Richest	61.3		1.84**(1.15–2.95)
**Place of residence**		< 0.0001	
Urban	43.7		0.81*(0.64–1.03)
Rural	28.7		
**Division**		< 0.0001	
Barisal	24.5		
Chittagong	26.0		0.55***(0.36–0.85)
Dhaka	42.7		1.12(0.72–1.76)
Khulna	42.7		1.27(0.8 -2)
Mymensingh	26.1		1.25(0.78–2.02)
Rajshahi	35.6		1.05(0.66–1.67)
Rangpur	27.8		0.64*(0.4–1.01)
Sylhet	22.6		1.19(0.74–1.9)
**Total**	**32.7**		

***p < 0.0001; **p < 0.05; *p < 0.10; p-values are based on chi-square test. AOR: Adjusted odds ratio; CI: Confidence interval

Table 3 presents the results of multilevel logistic regression performed to check for the cluster effect on variables in the study. Surprisingly, the results do not change drastically. The odds from the multilevel analysis mostly remain same with some slightly higher odds in case of age (≥ 35 years), ANC (4 or more visits) and place of delivery (private) and wealth index (richest).

**Table 3:** Odds ratio of caesarean birth among women aged 15-49 in Bangladesh by background characteristics, Bangladesh Demographic Health Survey, 2017-18

Background characteristics	AOR [95% CI]
**Women’s age (in years)**	
15–24	Ref.
25–34	1.18(0.89–1.57)
≥35	2.12**(1.20–3.75)
**Women’s education**	
No education	Ref.
Primary	0.57*(0.30–1.09)
Secondary	0.62(0.33–1.18)
Higher	0.71(0.35–1.42)
**Working status**	
No	Ref.
Yes	0.77**(0.61–0.98)
**Mass Media Exposure**	
No Exposure	Ref.
Exposure	1.27*(0.96–1.67)
**Body Mass Index**	
Underweight	0.98(0.72–1.33)
Normal	Ref.
Overweight and Obese	1.42***(1.09–1.85)
**Birth order**	
First	Ref.
2nd order	1.07(0.80–1.41)
3 or more	0.72*(0.49–1.04)
**ANC visits**	
No visit	Ref.
1–3 visits	1.69(0.77–3.72)
4 or more	2.51**(1.13–5.56)
**Place of delivery**	
Public	Ref.
Private	7.36***(5.74–9.42)
**Husband’s education**	
No education	
Primary	0.72(0.47–1.10)
Secondary	0.76(0.49–1.17)
Higher	0.94(0.57–1.55)
**Husband’s occupation**	
Unemployed	Ref.
Professional/Technical/Managerial	1.28(0.58–2.84)
Sales	1.15(0.55–2.42)
Agricultural	1.33(0.62–2.84)
Services	1.55(0.73–3.3)
Skilled And Unskilled	1.13(0.55–2.34)
**Religion**	
Islam	Ref.
Others	0.94(0.66–1.35)
**Wealth Index**	
Poorest	Ref.
Poorer	1.27(0.85–1.92)
Middle	1.22(0.80–1.86)
Richer	1.08(0.70–1.68)
Richest	1.90**(1.15–3.13)
**Place of residence**	
Urban	0.81(0.62–1.06)
Rural	Ref.
**Division**	
Barisal	Ref.
Chittagong	0.54**(0.33–0.86)
Dhaka	1.12(0.69–1.82)
Khulna	1.26(0.77–2.09)
Mymensingh	1.26(0.74–2.12)
Rajshahi	1.05(0.63–1.74)
Rangpur	0.63*(0.38–1.04)
Sylhet	1.16(0.69–1.96)
**ICC (S.E.)**	**7.1 (0.04)**

Ref.-Reference category; AOR: Adjusted odds ratio; CI: Confidence interval; ICC: Intra-class correlation coefficient; S.E.-Standard error

### Results from decomposition analysis for the contribution of various explanatory variables to caesarean births among women aged 15–49 years

The study decomposed the concentration index to explore the contribution of different socioeconomic variables to inequalities in caesarean deliveries. Decomposition results (i.e. elasticity, concentration index, absolute contribution, and per cent contribution) are presented in Table 4. The positive scores of the concentration index denote that caesarean deliveries concentrated among rich women for that particular predictor and vice-versa. Women aged ≥ 35 years, working and had three or more birth orders were concentrated amongst the bottom segment of the population regarding inequalities in caesarean births. While women who had mass media exposure, overweight/obese, received four or more ANC, delivered birth in a private health facility, and those who belonged to richest families were concentrated in the upper segment of the population in case of inequalities in caesarean births. Place of delivery, household wealth status, ANC visits, body mass index, women’s educational status and mass media exposure contributed the maximum to the inequality in caesarean births. About 65% of inequality was explained by place of delivery followed by wealth status of the household (about 13%). ANC visits explained about 5%. Furthermore, the BMI status of the women had a significant contribution to caesarean births-related inequality (4%). About 4% of the inequalities in caesarean births were explained by mass media exposure, followed by working status (about 2%).

**Table 4:** Effect and contribution of predictor variables of caesarean delivery among women aged 15-49 in Bangladesh based on decomposition analysis, Bangladesh Demographic Health Survey, 2017-18

Background characteristics	Elasticity	CCI	Absolute contribution	% contribution	
**Women’s age (in years)**					
15–24					
25–34	0.005	0.023	0.000	0.12	
≥35	0.003	-0.017	0.000	-0.06	0.07
**Women’s education**					
No education					
Primary	-0.012	-0.272	0.003	3.68	
Secondary	-0.009	0.046	0.000	-0.44	
Higher	0.002	0.445	0.001	0.97	4.21
**Working status**					
No					
Yes	-0.009	-0.174	0.002	1.76	1.76
**Mass Media Exposure**					
No Exposure					
Exposure	0.017	0.207	0.004	3.82	3.82
**Body Mass Index**					
Underweight	0.002	-0.177	0.000	-0.37	
Normal					
Overweight and Obese	0.013	0.311	0.004	4.37	4.00
**Birth order**					
First					
2nd order	-0.002	0.030	0.000	-0.05	
3 or more	-0.011	-0.140	0.002	1.67	1.62
**ANC visits**					
No visit					
1–3 visits	0.012	-0.108	-0.001	-1.45	
4 or more	0.033	0.172	0.006	6.13	4.68
**Place of delivery**					
Public					
Private	0.226	0.262	0.059	64.67	64.67
**Husband’s education**					
No education					
Primary	-0.009	-0.210	0.002	2.13	
Secondary	-0.009	0.125	-0.001	-1.16	
Higher	-0.001	0.452	0.000	-0.49	0.48
**Husband’s occupation**					
Unemployed					
Professional/Technical/Managerial	0.001	0.551	0.001	0.78	
Sales	0.001	0.162	0.000	0.18	
Agricultural	0.002	-0.356	-0.001	-0.78	
Services	0.004	0.051	0.000	0.20	
Skilled And Unskilled	0.004	-0.039	0.000	-0.15	0.24
**Religion**					
Islam					
Others	0.002	-0.084	0.000	-0.20	-0.20
**Wealth Index**					
Poorest					
Poorer	0.003	-0.378	-0.001	-1.03	
Middle	0.002	0.021	0.000	0.05	
Richer	0.004	0.412	0.001	1.57	
Richest	0.014	0.807	0.012	12.66	13.25
**Place of residence**					
Urban	0.000	0.398	0.000	0.00	0.00
Rural					
**Division**					
Barisal					
Chittagong	-0.010	0.072	-0.001	-0.79	
Dhaka	0.006	0.274	0.002	1.88	
Khulna	0.004	0.022	0.000	0.11	
Mymensingh	0.001	-0.222	0.000	-0.34	
Rajshahi	0.002	-0.076	0.000	-0.13	
Rangpur	-0.003	-0.330	0.001	1.04	
Sylhet	0.003	-0.124	0.000	-0.35	1.41
**Calculated CCI**			**0.092**		
**Actual CCI**			**0.270**		
**Residual**			**0.178**		

**Fig. 2:**
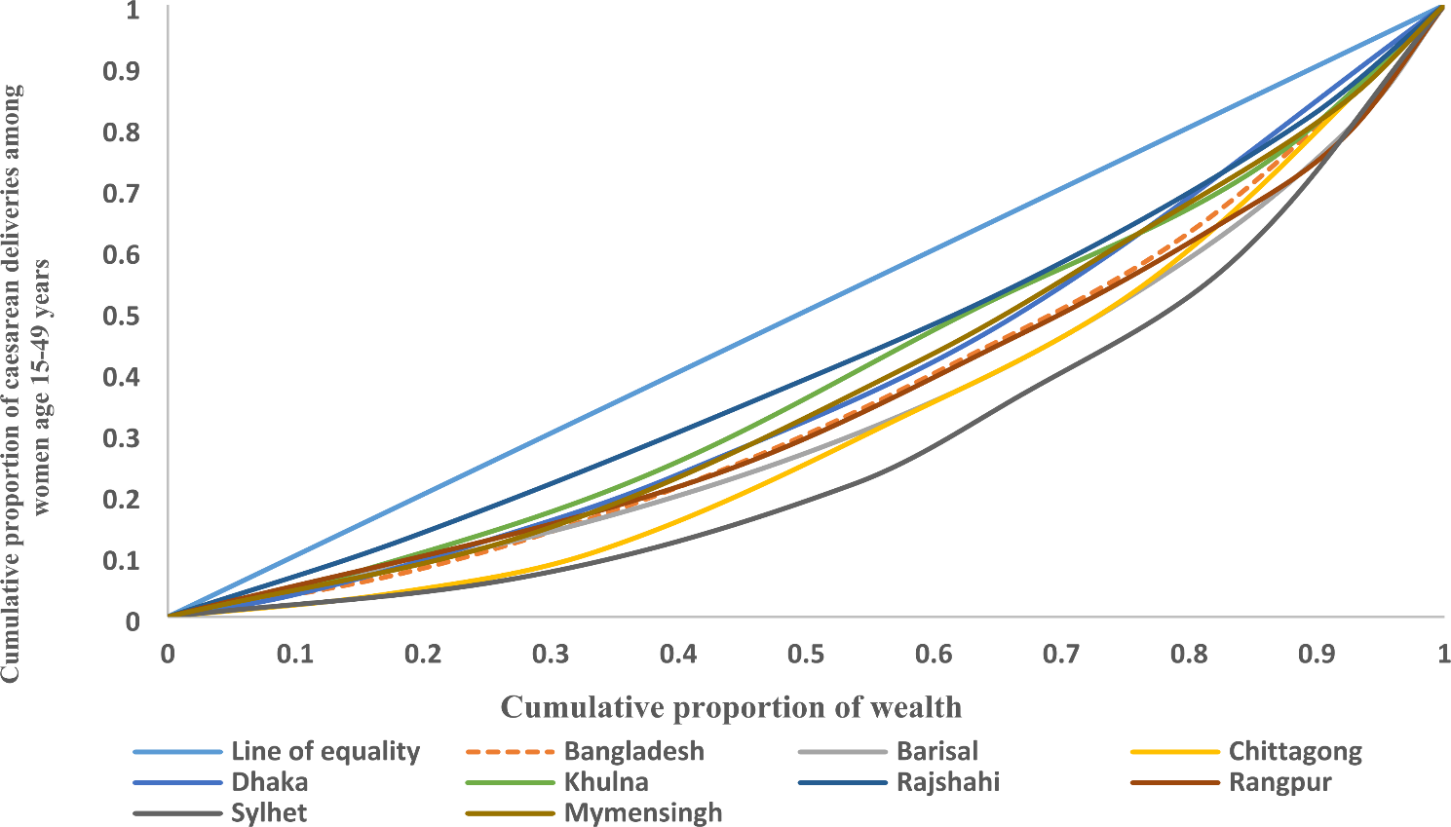
Concentration curve for inequality in caesarean section deliveries among women in Bangladesh and its divisions, Bangladesh Demographic Health Survey, 2017-18

Figure 2 shows the curves plotted to depict the inequality in caesarean section deliveries in Bangladesh and its divisions. The curve for Bangladesh was below the line of equity which meant that the instances of caesarean section deliveries were concentrated among the richest population. Similarly, the concentration curve for every division of Bangladesh lies down the line of equality, portraying an inequality in the caesarean section.

## Discussion

In the present study, we analyzed the prevalence of C-section deliveries and its significant determinants among women aged 15–49 in Bangladesh. The study reported that about one-third of women delivered by C-section, which was way higher than the upper limit of the WHO critical threshold of C-Section (15%) for any country [13]. Past studies from Bangladesh have shown an improved condition of women’s education and medical facilities in small towns and villages [27, 31]. Almost 24% prevalence of CS delivery was observed in Bangladesh by a study conducted in 2019. Our study has shown an increased prevalence of CS delivery in Bangladesh but the important risk factors and their individual contribution align with the previous study [32].

There has been a serious and commendable drive to uplift the maternal and child health in Bangladesh, which has led to a significant decrement in maternal and child deaths. However, better-quality health facilities have also given rise to preventable C-section deliveries in Bangladesh. Given the amount of inequality in health care services in Bangladesh, it becomes important to have a clear understanding of the underlying causes of the observed inequality in health facility delivery, skilled birth assistance, and C-section delivery. Measuring and elucidating socioeconomic inequalities are important for not only health planners to design policies that target specific sub-groups of the population but also towards the monitoring and evaluation of progress towards goals 3.1 and 3.2 of the SDGs [33]. Social stratification or hierarchy is produced by various social determinants that cause the inequalities in health care services in the society. The findings of the present study exhibit that reduction of inequalities in these social determinants of health is crucial in order to increase the coverage of health care services among the women from the disadvantaged sections of the society [19]. The present study revealed that the socioeconomic factors and health-related factors played an important role in C-section deliveries among Bangladeshi women. The study’s findings revealed that factors like age, education, wealth, employment status, media exposure, birth order, nutritional status, number of ANC visits, delivery in health facilities are significant predictors of caesarean deliveries in Bangladesh [23]. Women from higher age groups were more likely to be delivered through C-sections. This finding was also supported by other studies that documented advanced age as an important factor behind C-section deliveries [34]. One surprising finding of this study was that C-section delivery rates were found low among educated women. On contrary to this, previous studies have revealed that educated and wealthy women have more births though a C-section delivery [2]. One possible explanation for this is that being educated and wealthy enables a woman to access the best facilities, afford good private hospitals for delivery, and make her own decisions about getting a C-section delivery [35]. Studies have revealed that a woman nowadays does not want a vaginal delivery due to fear of labour pain and other consequences and opts for C-section deliveries. They might be a reason for the increment in CS delivery in Bangladesh among educated and wealthy women [23]. In the South and South-East Asian countries, place of delivery emerges as an important factor to influence the choice to undergo a caesarean section delivery [36]. Likewise, the results of the present study reveal that the caesarean deliveries were significantly associated with ANC visits among women and those delivered to a private health facility. Women with more ANCs were more likely to deliver by caesarean delivery in Bangladesh. Similar was the case with women who delivered their babies in the private health care facility. Regardless of public health facilities being accessible to everyone, reasons corresponding to lack of awareness, unavailability of specific services and high out of pocket payment regarding health care impact decision of women to choose private maternal care services over public facilities [20]. Previous works have shown that ANC services received from deliveries performed in private facilities enhance the chance of CS. Reasons from the past literature show that the profit-driven private facilities take up this chance and contribute to increasing CS without proper indications for this procedure [2].

Birth order and caesarean delivery have often shown a negative association. The present study found that caesarean delivery probability was higher among women with first birth orders. This finding goes in tune with a study concerning nine developing countries of South and South-East Asia namely, Vietnam, India, Maldives, Timor-Leste, Nepal, Indonesia, Pakistan, Bangladesh, and Cambodia which revealed a same picture of birth order being significantly associated with caesarean delivery [15]. Women who were working were found to be at lower risk of delivering through a caesarean section. Studies in the past on Bangladesh have also shown similar results; a possible explanation could be that working women undergo time constraints because of their work, which reduces their opportunities for receiving antenatal care [23, 37].

Further, the present study found that women exposed to mass media showed a higher preponderance of having a baby delivered through the caesarean method. Advances in mass media like TV, social media applications, newspaper, radio and their usage have created awareness among women [2]. A multi country analysis addressing the community factors affecting caesarean section rates revealed that regular use of traditional media (TV, radio and newspaper) and maintaining social networks have an impact on the risks of a caesarean section birth [38]. One of the risk factors associated with caesarean deliveries was the BMI of the mother. It was found that overweight/ obese women were more likely to have a C-section performed compared to those with a normal BMI. Results from a prospective population-based cohort study in Norway aligns with the findings of the present study that the performance of C-sections depends on the obesity of the mother [39].

The study also captured the existing socioeconomic inequality in caesarean deliveries in Bangladesh utilizing the latest BDHS data. A noteworthy amount of variation was observed among the divisions of the country. Factors like place of delivery, household wealth status, ANC visits, body mass index, women’s education and mass media contributed the most to the inequality in caesarean births in Bangladesh. The highest contributor to inequality was the place of delivery, followed by wealth status and ANC visits. The concentration graph reflected that the instances of caesarean section deliveries were concentrated among the wealthiest population, which adds more value to the already discussed point that caesarean deliveries were more frequent among the wealthy women of Bangladesh.

Despite these strengths, there are some potential limitations to the current study. First, because the survey does not include household income and expenditure information, the study employed a wealth index to calculate socioeconomic inequality, an asset-based wealth index as a proxy for household SES. Second, the study considered all probable factors impacting caesarean delivery available in the survey; nevertheless, there may be other factors at play that can stimulate the instances of caesarean delivery among women in Bangladesh. Third, the body mass index is calculated based on the information on women’s height and weight that were measured during the survey. However, the information on delivery methods was collected for the three years preceding the survey. Since BMI is not stable over time, this may partially affect the observed association. Lastly, since the data were cross-sectional, no causal inferences can be formed.

## Conclusion

Caesarean delivery among women of reproductive age 15–49 have tremendously increased in Bangladesh and its divisions. The prevalence of caesarean deliveries is 32% which is way above the ideal rate of 15% of caesarean delivery recommended by the World Health Organization. Socioeconomic factors like age, education, wealth, media and working status play an important role in predicting caesarean delivery rates in Bangladesh. Moreover, factors like obesity, birth order, and ANC visits emerged as important factors of caesarean delivery. Lastly, socioeconomic inequality prevails in the caesarean deliveries in Bangladesh. The place of delivery, household wealth status, ANC visits, body mass index, women’s education and mass media have been the highest contributors to the inequality. The present study revealed that these factors might be considered important for reducing C-section rates in Bangladesh. The study, through its findings, suggests that the health authorities should intervene, formulate specialized programs and spread awareness about the ill effects of caesarean deliveries amongst the most vulnerable groups of women in Bangladesh. The authorities should provide awareness to pregnant women on every comprehensive checkup about the pros and cons of C-section delivery and how to maintain good physical health during their pregnancy period.

## Data Availability

The data is available online on the official website of the Demographic Health Survey, therefore, the data can be assessed through https://www.dhsprogram.com/data/available-datasets.cfm.

## List of abbreviations

BDHS: Bangladesh Demographic Health Survey
AOR: Adjusted Odds Ratio
CI: Confidence Interval
WHO: World Health Organization
ANC: Antenatal Care
BMI: Bio Mass Index
CS: Caesarean Section
NIPORT: National Institute for Population Research and Training
EAS: Enumeration Areas
BBS: Bangladesh Bureau of Statistics
PSU: Primary Sampling Unit
HIV/AIDS: Human Immuno Deficiency Virus/ Acquired Immune Deficiency Syndrome
CCI: Concentration Index
CC: Concentration Curve
SES: Socioeconomic Status
